# Editorial: Special Issue — Enzyme Immobilization

**DOI:** 10.3390/molecules191220671

**Published:** 2014-12-10

**Authors:** Roberto Fernandez-Lafuente

**Affiliations:** Institute of Catalysis and Petrochemistry-CSIC, Campus UAM-CSIC Cantoblanco, Madrid 28049, Spain; E-Mail: rfl@icp.csic.es; Tel.: +34-91-585-4941; Fax: +34-91-585-4760

Immobilization of enzymes and proteins is a requirement for many industrial enzyme applications, as this facilitates enzyme recovery and reuse. Bearing in mind this necessity, the coupling of immobilization to the improvement of other enzyme features has been pursued by many researchers, and nowadays immobilization is recognized as a tool to improve not only stability, but also enzyme selectivity, specificity, resistance to inhibition or chemical modifiers, *etc.* To achieve these overall improvements of enzymes’ properties via immobilization, it is necessary to both develop new immobilization systems suitable for these purposes, and to achieve a deeper knowledge of the mechanisms of interaction between enzymes and activated solids. That way, immobilization of enzymes, far being an old-fashioned methodology to just reuse these expensive biocatalysts, is a tool of continuous interest that requires a continuous effort to be exploited in all its potential. This special issue collects 23 papers reporting advances in the field of immobilization of enzymes.

Several of the papers included on this special issue are very interesting reviews on the cutting edges of the techniques used and the future of enzyme immobilization. Thus, using oxidoreductases as a model enzyme, the possibilities of improving enzyme properties via immobilization [1] and the opportunities that inorganic materials [2] or magnetic nanoparticles provide for enzyme immobilization [3] have been reviewed. In another review, the fusion of the enzyme of interest to polyhydroxyalkanoate with covalently attached synthase is discussed as a method to achieve site-directed immobilization [4]. Reviews also include the design of enzymatic biosensors for drug screening and pharmaceutical kinetic studies [5].

Some interesting new materials for enzyme immobilization have been also proposed. For example, tailor-made siliceous ordered mesoporous materials have been used for laccase immobilization [6], methacrylate-substituted polyphosphazene beads has been used for immobilization of lipase from *Candida rugosa* [7], hydrophobic core-shell supports have been used to immobilize lipase B from *Candida antarctica* [8], inulinase has been non-covalently immobilized on carbon nanotubes [9], mixed-function-grafted mesoporous silica gel support has been used to immobilize the lipase from *Burkholderia cepacia* by hydrophobic adsorption and covalent attachment [10], poly(ethylene glycol) decorated polystyrene nanoparticles modified by the adsorption of Congo red was used for immobilization of lipases [11], organic/magnetic nanocarriers bearing hyperbranched poly(amido acid)s were used to immobilize γ*-*glutamyltranspeptidase [12], wool activated by cyanuric chloride has been used to immobilize α-amylase [13], laccase has been immobilized on a pan/adsorbents composite nanofibrous membrane [14], and styrene-divinylbenzene beads have been evaluated to immobilize lipases [15,16].

In some instances, the emphasis of the papers has been a focus on the improved applications of the immobilized enzymes for a specific process. Covalently immobilized lipase from *Rhizopus oryzae* on sepiolite was used in the production of new biofuel similar to biodiesel [17]. Horseradish peroxidase immobilized via glutaraldehyde chemistry was used in the degradation of 2,4-dichlorophenol [18]. Nucleoside 2'-deoxyribosyltransferase has been produced and immobilized onto different supports with the objective of stabilizing its multimeric structure, this has enabled the synthesis of nucleoside 2'-deoxyadenosine from 2'-deoxyuridine and adenine [19]. The immobilized thermophilic esterase from *Archaeoglobus fulgidus* was adsorbed on hydrophobic Sepabeads EC-OD and further treated with glutaraldehyde and successfully employed in the synthesis of poly(ε-caprolactone) [20]. Lecitase immobilized on styrene-divinylbenzene beads has been evaluated in the synthesis of flavor esters under ultrasound stirring conditions [15]. Changes in enzyme specify upon immobilization are discussed in several papers [16,21].

Development of techniques to visualize the immobilized enzymes is a technique that may improve the control and understanding of this process. This was the main matter of [22], where confocal microscopy was used so the authors could identify the distribution of enzymes trapped in alginate. Finally, the use of immobilized enzymes for the development of implantable glutamate sensors is the subject of the last of the contributions of this special issue [23].

All contributions help provide a vision of the current and future trends in the development of enzyme immobilization and biocatalysis. We hope that this special issue may help to understand the great potential of enzyme immobilization to solve enzyme limitations and encourage future research on this matter, perhaps the topic of future new special issues on the subject.

## References

[1] Guzik U., Hupert-Kocurek K., Wojcieszyńska D. (2014). Immobilization as a strategy for improving enzyme properties-application to oxidoreductases. Molecules.

[2] Zucca P., Sanjust E. (2014). Inorganic materials as supports for covalent enzyme immobilization: Methods and mechanisms. Molecules.

[3] Xu J., Sun J., Wang Y., Sheng J., Wang F., Sun M. (2014). Application of iron magnetic nanoparticles in protein immobilization. Molecules.

[4] Hooks D.O., Venning-Slater M., Du J., Rehm B.H.A. (2014). Polyhydroyxalkanoate synthase fusions as a strategy for oriented enzyme immobilization. Molecules.

[5] Gonçalves A.M., Pedro A.Q., Santos F.M., Martins L.M., Maia C.J., João A., Queiroz J.A., Passarinha L.A. (2014). Trends in protein-based biosensor assemblies for drug screening and pharmaceutical kinetic studies. Molecules.

[6] Gascón V., Díaz I., Márquez-Álvarez C., Blanco R.M. (2014). Mesoporous silicas with tunable morphology for the immobilization of Laccase. Molecules.

[7] Qian Y.-C., Chen P.-C., He G.-J., Huang X.-J., Xu Z.-K. (2014). Preparation of polyphosphazene hydrogels for enzyme immobilization. Molecules.

[8] Pinto M.C.C., Freire D.M.G., Pinto J.C. (2014). Influence of the morphology of core-shell supports on the immobilization of lipase B from *Candida antarctica*. Molecules.

[9] Garlet T.B., Weber C.T., Klaic R., Foletto E.L., Jahn S.L., Mazutti M.A., Kuhn R.C. (2014). Carbon nanotubes as supports for inulinase immobilization. Molecules.

[10] Abaházi E., Boros Z., Poppe L. (2014). Additives enhancing the catalytic properties of lipase from burkholderia cepacia immobilized on mixed-function-grafted mesoporous silica gel. Molecules.

[11] Silva R.A., Carmona-Ribeiro A.M., Petri D.F.S. (2014). Catalytic behavior of lipase immobilized onto congo red and PEG-Decorated particles. Molecules.

[12] Juang T.-Y., Kan S.-J., Chen Y.-Y., Tsai Y.-L., Lin M.-G., Lin L.-L. (2014). Surface-functionalized hyperbranched poly(amido acid) magnetic nanocarriers for covalent immobilization of a bacterial γ-Glutamyltranspeptidase. Molecules.

[13] Mohamed S.A., Khan J.A., Al-Bar O.A.M., El-Shishtawy R.M. (2014). Immobilization of trichoderma harzianum α-Amylase on treated wool: Optimization and characterization. Molecules.

[14] Wang Q., Cui J., Li G., Zhang J., Li D., Huang F., Wei Q. (2014). Laccase immobilized on a pan/adsorbents composite nanofibrous membrane for catechol treatment by a biocatalysis/adsorption process. Molecules.

[15] Alves J.S., Garcia-Galan C., Schein M.F., Silva A.M., Barbosa O., Ayub M.A.Z., Fernandez-Lafuente R., Rodrigues R.C. (2014). Combined effects of ultrasound and immobilization protocol on butyl acetate synthesis catalyzed by CALB. Molecules.

[16] Garcia-Galan C., Barbosa O., Hernandez K., Dos Santos J.C.S., Rodrigues R.C., Fernandez-Lafuente R. (2014). Evaluation of styrene-divinylbenzene beads as a support to immobilize. Molecules.

[17] Luna C., Verdugo C., Sancho E.D., Luna D., Calero J., Posadillo A., Bautista F.M., Romero A.A. (2014). Biocatalytic behaviour of immobilized *Rhizopus oryzae* lipase in the 1,3-selective ethanolysis of sunflower oil to obtain a biofuel similar to biodiesel. Molecules.

[18] Chang Q., Tang H. (2014). Immobilization of horseradish peroxidase on NH_2_-modified magnetic Fe_3_O_4_/SiO_2_ particles and its application in removal of 2,4-dichlorophenol. Molecules.

[19] Fresco-Taboada A., Serra I., Fernández-Lucas J., Acebal C., Arroyo M., Terreni M., de La Mata I. (2014). Nucleoside 2'-deoxyribosyltransferase from psychrophilic bacterium Bacillus psychrosaccharolyticus—Preparation of an immobilized biocatalyst for the enzymatic synthesis of therapeutic nucleosides. Molecules.

[20] Wang M., Shi H., Wu D., Han H., Zhang J., Xing Z., Wang S., Li Q. (2014). Glutaraldehyde cross-linking of immobilized thermophilic esterase on hydrophobic macroporous resin for application in poly(ε-caprolactone) synthesis. Molecules.

[21] Skoronski E., Fernandes M., de Lourdes Borba Magalhães M., da Silva G.F., João J.J., Lemos Soares C.H., Fúrigo A.F. (2014). Substrate specificity and enzyme recycling using chitosan immobilized laccase. Molecules.

[22] Tsai C.-T., Meyer A.S. (2014). Enzymatic cellulose hydrolysis: Enzyme reusability and visualization of β-Glucosidase immobilized in calcium alginate. Molecules.

[23] Tseng T.T.-C., Chang C.-F., Chan W.-C. (2014). Fabrication of implantable, enzyme-immobilized glutamate sensors for the monitoring of glutamate concentration changes *in vitro* and *in vivo*. Molecules.

